# Rapid Fabrication of Large-Area Concave Microlens Array on ZnSe

**DOI:** 10.3390/mi12040458

**Published:** 2021-04-19

**Authors:** Fan Zhang, Qing Yang, Hao Bian, Xun Hou, Feng Chen

**Affiliations:** 1 State Key Laboratory for Manufacturing System Engineering, School of Electronic Science and Engineering, Xi’an Jiaotong University, Xi’an 710049, China; zf19900625@stu.xjtu.edu.cn (F.Z.); houxun@mail.xjtu.edu.cn (X.H.); 2 School of Mechanical Engineering, Xi’an Jiaotong University, Xi’an 710049, China; yangqing@mail.xjtu.edu.cn

**Keywords:** infrared optics, femtosecond laser, microlens array, ZnSe

## Abstract

A rapid and single-step method for the fabrication of a zinc selenide (ZnSe) concave microlens array through the high-speed line-scanning of a femtosecond laser pulse is presented. Approximately 1.1 million microlenses, with minimized volume and high transparency at wavelengths between approximately 0.76–20 μm were fabricated within 36 min. More importantly, the size of the microlenses can be controlled by adjusting the laser power. Their high-quality infrared optical performance was also demonstrated. This method holds great promise for the development of ZnSe-based micro-optical devices.

## 1. Introduction

Microlens arrays (MLAs) have diverse applications for infrared detection [1], beam homogenization [2], microimaging [3], optical communication [4,5], and microfabrication [6]. At present, silicon and quartz are widely used for processing MLAs. However, the infrared cutoff wavelength of quartz is shorter (the longest wavelength is approximately 3.5 μm). In the very important atmospheric transparent second (3–5 μm) and third (8–12 μm) windows, quartz material cannot transmit light, and silicon and quartz are limited in nonlinear applications owing to their low nonlinear characteristics [7,8].

As an important II–VI semiconductor, zinc selenide (ZnSe) was one of the earliest discovered semiconductors, and is probably one of the most critical electronic and optoelectronic materials, with outstanding applications in nonlinear devices [9]. Compared with silicon and quartz, ZnSe has an excellent medium and far infrared transmittance (0.5–22 μm), a high refractive index (2.3–2.5), and a high nonlinear refractive index (8.3 × 10^−18^ m^2^/W) [10,11]. ZnSe has good mechanical properties, high thermal conductivity, and is insoluble in water. It cannot be ruptured under normal mechanical pressure or impact. Due to its excellent chemical and physical properties, ZnSe has been widely used in solar cells, lasers, logic gates, flat panel displays and transistors [12,13,14,15]. Recently, ZnSe has garnered tremendous interest because of its valuable potential for both basic physical research and applications in micro-nano component construction. 

ZnSe is relatively easy to process as a soft crystal. At present, manufacturing techniques have been developed, including lacquer disk technology, traditional grinding and polishing, and single-point diamond turning [16,17]. Despite tremendous progress, there are still some limitations among these techniques, such as high time consumption, high process complexity, and difficulty in consistency control [18]. As an appealing alternative, femtosecond laser manufacturing has become a powerful tool for processing optical devices based on a wide range of materials, including semiconductors [19], metals [20], and dielectrics [21]. Earlier reports have already proven the effectiveness of the femtosecond laser ablation and etching technologies for fabricating optical devices [22,23,24]. However, the etching process involved in this technology still takes a lot of time.

In this work, we propose a rapid process for the realization of large-area concave infrared MLAs that addresses the limitations of traditional technologies. Our method, a rapid single-step process, exploits a high-speed femtosecond laser scanning technology. Each microlens can be formed by a femtosecond laser pulse. Within 36 min, approximately 1.1 million microlenses can be fabricated. The ablation crater of ZnSe can directly form a concave microlens under laser irradiation. The range of laser power is discussed, and we conclude how the uniformity of the structures is achieved. Furthermore, this article shows the three-dimensional (3D) morphology, as well as the IR optical properties, of the MLAs.

## 2. Materials and Methods

**Figure 1:**
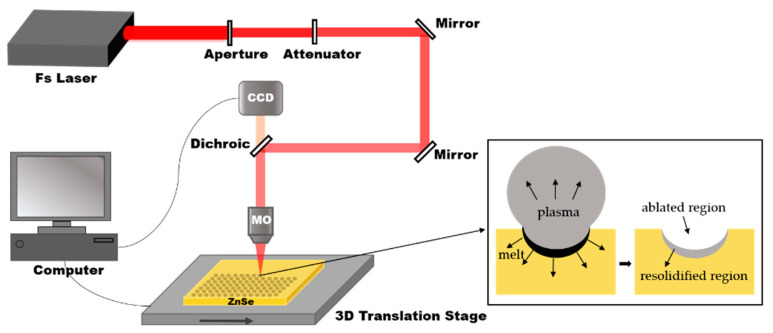
A schematic diagram of the fabrication process. The inset shows the formation mechanism of a microlens irradiated by a single femtosecond laser pulse, in which the Fs Laser is the femtosecond laser, CCD is imaging sensor, MO is the microscope objective.

The IR ZnSe MLAs manufacturing process includes only one step: a fast femtosecond laser scanning process, as shown in Figure 1. In the present experiment, a commercial ZnSe sample (II–VI Incorporated, Pittsburgh, PA, USA) was used. The ZnSe was installed on the 3D translation stage (Prior Scientific, Cambridge, England, 10 ≤ v ≤ 20 mm/s ) after ultrasonic cleaning in deionized water for 10 min and drying in ambient atmosphere. The laser was generated from a regenerative, amplified Ti: sapphire laser system (Coherent Libra-usp-he; pulse duration: 50 fs; central wavelength: 800 nm; repetition rate: 1 kHz). Different optical density filters can change the pulse energy, which can be set to 5–30 mW. The laser beam was focused on the ZnSe surface through a 0.45 NA, 20X objective lens (Nikon). The femtosecond laser exposure spots were generated point-by-point by the pulsed laser system. A parabolic microlens was formed on the surface of the ZnSe due to the Gaussian distribution of the femtosecond laser beam and the unbalanced energy deposition of the laser pulses to the sample. In the fast-scanning process, the average distance between the adjacent microlenses was adjusted by the transverse scanning speed. For example, the sample was translated in a direction perpendicular to the laser beam at a speed of 12 mm/s; the microlenses, with a separation space of 12 μm, were generated at a laser repetition rate of 1 kHz per second. 

Scanning electron microscopy (SEM) images of the fabricated microlenses were observed using a Flex 1000 scanning electron microscope (Hitachi, Tokyo, Japan). A LEXT-OLS4000 laser confocal microscope (Olympus, Japan) (Olympus, Tokyo, Japan) was used to obtain the three-dimensional topography and the corresponding profile curves.

## 3. Results and Discussion

**Figure 2:**
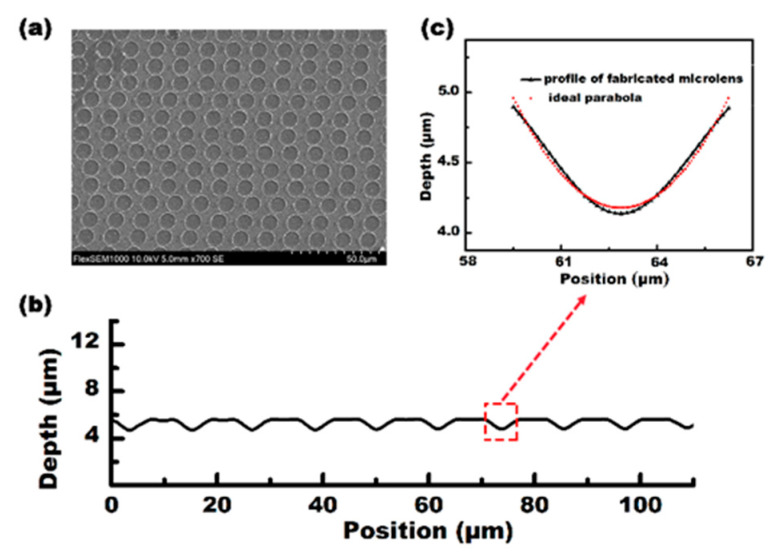
The characterization of the quasi-periodic microlens array (MLA) on a ZnSe surface. (**a**) A top-view scanning electron microscopy (SEM) image of the MLAs; (**b**) the actual profile of the microlens (line) and the ideal parabolic profile (dots); (**c**) A cross-section profile of the MLA that is shown in (**a**).

With the scanning speed set at 15 mm/s and the pulse energy set at 5 mW quasi-periodic microlenses, with an average size of 15 μm, could be observed, and 1000 microlenses were generated per second at a laser repetition rate of 1 kHz. The processing efficiency was 1 × 10^5^ times higher than that of the femtosecond laser direct writing method [25]. Figure 2a shows the SEM image of the MLA. Figure 2b shows the cross-sectional profile of several microlenses measured along the scanning direction. The measurement results show that the average diameter (D) of the IR microlens is 6.65 μm, and the depth (h) is 0.804 μm. Moreover, according to the measurements and the theoretical fitting data, as shown in Figure 2c, the microlenses have a parabolic shape. Furthermore, the radius of the curvature (R), the focal length (f), and the numerical aperture (NA) of the microlenses with a parabolic surface are calculated as:

R = D^2^/8h, f = R/(n − 1), NA = D/2f (1)

where h is the sag height of the microlens, n = 2.63 is the refractive index of ZnSe, and D is the diameter of the microlens. Given D = 6.65 μm and h = 0.804 μm, we have R = 6.87 μm, f = 4.21 μm, and NA = 0.789.

The shape of the single-shot ablation areas shows a smooth crater surrounded by an elevated rim. The formation mechanism of a microlens is presented by analogy with Ben-Yakar’s theory. The major advantages of the femtosecond laser include its ability to produce a very high peak intensity and its rapid deposition of energy into the material. The high peak intensities allow energy to be transferred to the high band-gap materials through nonlinear processes. The rapid absorption of energy results in effective material removal before significant thermal diffusion to the substrate. Consequently, when the fluence of an incidental single femtosecond laser pulse exceeds the damage threshold of the ZnSe material, part of the absorbed laser energy remains in most of the materials and melts a thin layer below the ablated volume. When the temperature of the melt drops below the melting temperature, the melt will re-solidify. As shown in the illustration in Figure 1, the high-pressure plasma produces a pressure-driven, fluid movement, which makes the molten material flow outward from the center of the crater and forms a convex edge around the melted crater. This crater has a good concave, curved shape, thus forming an original microlens. The mechanical pressure exerted by the spherical plasma shock wave onto the molten ZnSe can make the surface of the concave crater smoother. In this way, a microlens can be rapidly fabricated through the rapid scanning process.

**Figure 3:**
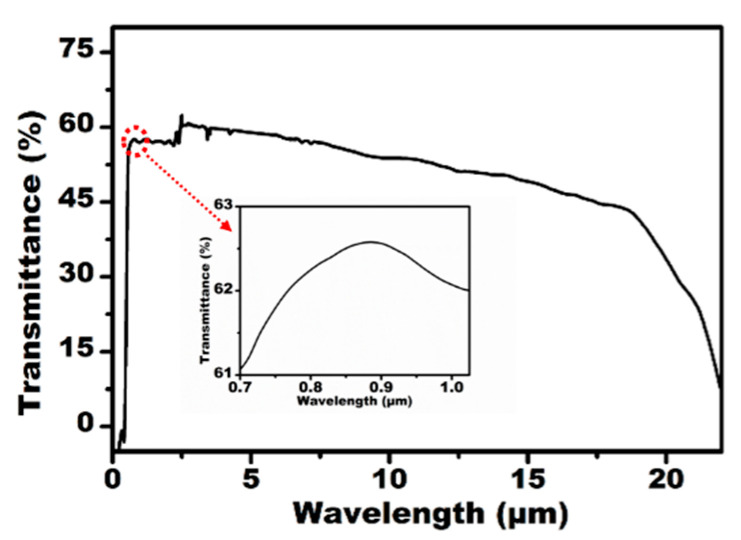
The measured transmittance of the ZnSe MLA.

**Figure 4:**
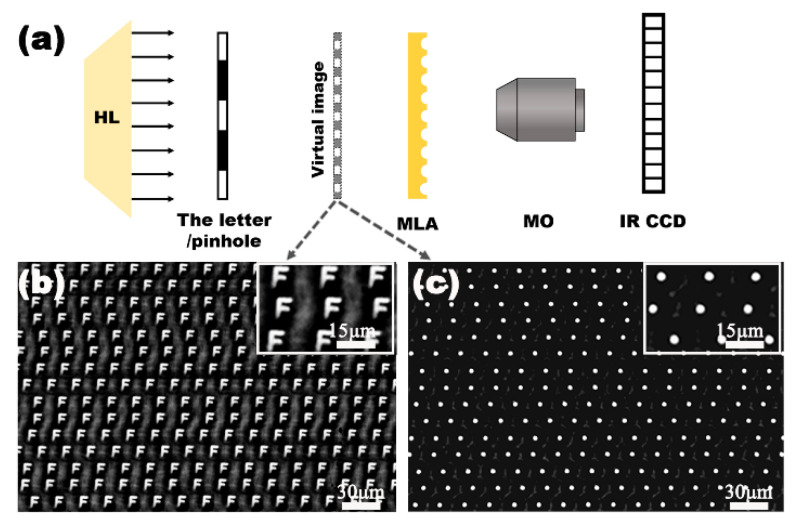
The IR imaging performance of the quasi-periodic MLA. (**a**) The simplified setups of the IR optical system for measurement, HL is the halogen lamp, MLA is the sample device, and MO is the microscope objective; IR CCD is the imaging sensor responsing in the IR region; (**b**) the false images of the letter “F”, using the laser power of 5 mW; (**c**) the false bright focal point of the fabricated MLA.

As an IR optical device, the transparency of the IR band and the visible band is the key characteristic for their practical applications. The transmittance of the fabricated IR MLA was measured using a Ultraviolet-Visible-Near infrared (UV-VIS-NIR) spectrophotometer (Lambda 750, Perkinelmer, Waltham, MA, USA) and Fourier transform infrared spectrometer (Nicolet iS 10, Thermo Fisher, Waltham, MA, USA), as shown in Figure 3. The IR MLAs show relatively high transmittance in the visible, near-infrared, and mid-infrared regions from 0.76 μm to 22 μm. Simultaneously, the IR MLA has been confirmed as a competitively arrayed IR imaging device using an IR optical microscope system. The IR microscope system is comprised of an illumination light (1500 nm), an IR charge-coupled device (CCD) camera (OW 1.7-CL-320, Raptor photonics, Northern Ireland, England), a three-dimensional translation stage, and an IR objective lens (Plan Apo, Nikon, Tokyo, Japan), as shown in Figure 4a. The letter “F” was chosen as the imaging target, as shown in Figure 4b. The clear image of “F” was uniform in size and shape, which indicated the uniformity of the structure and good IR imaging properties of the IR MLAs. When the letter was replaced by a pinhole, a clear IR image (Figure 4c) was captured. The brightness and the size of the spot-like virtual images were uniform. This shows that the IR MLAs have good consistency in their focal lengths.

**Figure 5:**
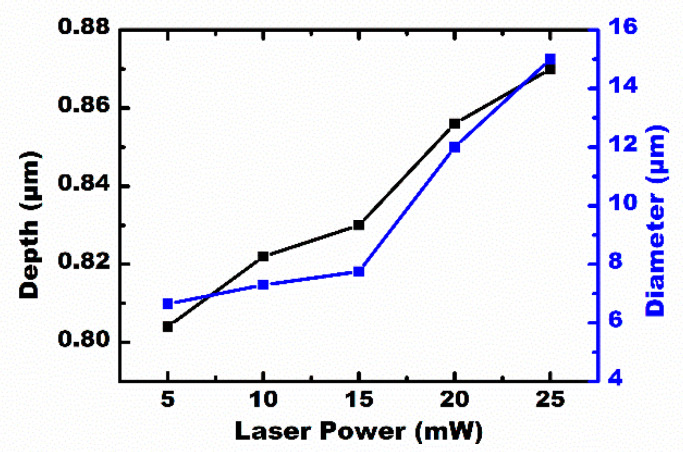
The dependencies of the diameter and depth of the concave microlens on laser power.

**Figure 6:**
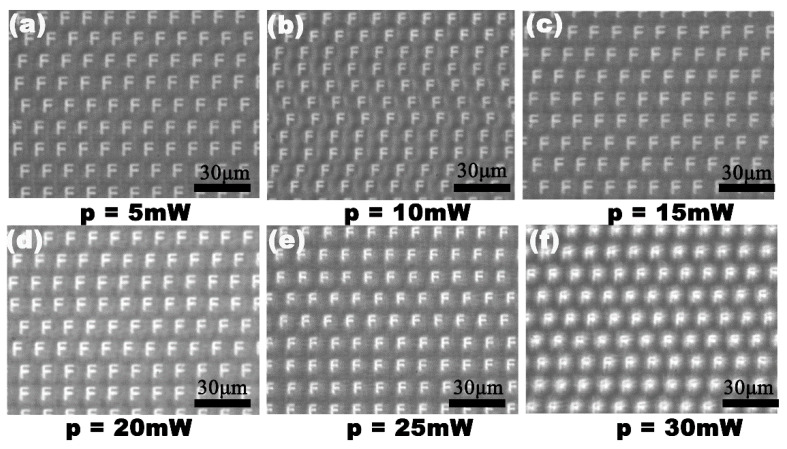
The IR optical performances of the MLAs fabricated at different laser power levels: (**a**) 5 mW; (**b**) 10 mW; (**c**) 15 mW; (**d**) 20 mW; (**e**) 25 mW; (**f**) 30 mW.

The scanning speed and the laser power are important parameters in this experiment. A series of microlenses, with a separation space of v/f μm, were created at a laser repetition rate of n kHz, moving the substrate at a speed of v mm/s. If the laser power was constant, n × 1000 microlenses were generated every second at a laser repetition rate of n kHz. However, the maximum speed of the three-dimensional translation stage becomes the only factor to affect the processing efficiency. During the process of laser irradiation, different profiles of the IR microlens could be obtained through a simple adjustment of the laser power. Figure 5 shows the dependence of the diameter, and the sag height of the formed IR microlens, on the laser power. The diameter and the height of the microlens were positively correlated with the laser power. The IR optical performances of the MLAs processed under the laser power of 5–30 mW are shown in Figure 6. The IR imaging performance is an important criterion used to measure the quality of the microlens. The image quality of the MLAs deteriorates with an increase in the laser power. When the laser power is higher than 25 mW, the rough micro/nano structures, formed by the high-power laser ablation [26,27], are only partially weakened by the smoothing process caused by the transient molten zone, so the rough ripple structure becomes obvious. Due to the scattering of incident light by the rough structure, the image quality of the IR MLA will degrade, as shown in Figure 6f. The results indicate that the most appropriate laser power range is 5 to 25 mW in our experiment.

## 4. Conclusions

In conclusion, a single-step fabrication for large-area IR MLAs was proposed. Approximately millions of microlenses could be easily fabricated on ZnSe within half an hour. In addition, maintaining a balance between the smoothing process induced by the transient melting zone and the large micro/nano structures formed by the high-power laser ablation is the key to the formation of high-quality MLAs. The most suitable laser power range is 5–25 mW. The diameter and the sag height of the microlens can be controlled by the laser power. Compared with the other method, our technology based on single femtosecond pulse irradiation, can rapidly fabricate the IR optical devices without wet etching. Approximately 1.1 million microlenses with minimized volume and high transparency at wavelengths of 0.7620 μm were fabricated in 36 min. Quantitative evaluation and imaging tests demonstrate good optical properties. In this paper, an effective method for the rapid fabrication of IR MLAs on ZnSe was proposed, which opens up a new method for the development of micro-optics based on ZnSe.

## Data Availability

The data that support the findings of this study are available from the corresponding author upon reasonable request.
